# Hepatobiliary surgery in the era of immunotherapy: integrating surgical timing, immune surveillance, and recurrence prevention

**DOI:** 10.3389/fimmu.2026.1865909

**Published:** 2026-07-01

**Authors:** Jingyi Xu, Lei Yang, Shuang Wang, Liusheng Wu, Yuehua Liang, Xialin Xie, Wenqiang Wang, Lu Gao, Jun Yan

**Affiliations:** 1 Department of Liver Surgery, Beijing Tsinghua Changgung Hospital, School of Clinical Medicine, Tsinghua Medicine, Tsinghua University, Beijing, China; 2 Department of Basic Medicine, Beijing Health Vocational College, Beijing, China

**Keywords:** biliary tract cancer, CtDNA, hepatobiliary surgery, hepatocellular carcinoma, immunotherapy, recurrence prevention

## 1 Introduction

Hepatobiliary surgery is entering a phase in which resectability is no longer purely an anatomic judgment. For hepatocellular carcinoma (HCC), intrahepatic cholangiocarcinoma (ICC), extrahepatic cholangiocarcinoma, and gallbladder cancer, decisions still depend on tumor extent, liver reserve, portal hypertension, biliary anatomy, nodal disease, performance status, and the feasibility of R0 resection. Yet immune checkpoint inhibitors (ICIs), anti-VEGF therapy, tyrosine kinase inhibitors (TKIs), and immunochemotherapy now influence whether, when, and how surgery should be performed (1, 2).

HCC provides the most mature perioperative immunotherapy signal. Nivolumab with or without ipilimumab, cemiplimab, and cabozantinib plus nivolumab have produced pathological regression before resection in selected patients (3–5), and deeper pathological response after neoadjuvant ICI therapy has been associated with improved relapse-free survival (6). Adjuvant data remain unsettled: IMbrave050 initially favored atezolizumab plus bevacizumab, updated follow-up weakened routine enthusiasm, and adjuvant sintilimab is supported by phase II rather than phase III evidence (7–9). Pre-transplant ICI exposure may aid downstaging but increases rejection risk when washout is short (10, 11).

Biliary tract cancer (BTC) is undergoing a parallel transition across ICC, perihilar cholangiocarcinoma, distal cholangiocarcinoma, and gallbladder cancer. TOPAZ-1 and KEYNOTE-966 established durvalumab or pembrolizumab plus gemcitabine-cisplatin for advanced BTC, while ICC studies of toripalimab, lenvatinib, and GEMOX suggest a conversion option in carefully selected patients (12–16). These data do not make perioperative immunotherapy routine for BTC, but they oblige surgeons to reassess R0 feasibility after response, manage drainage and sepsis during treatment, and preserve oncologically indicated nodal staging. This Opinion therefore uses HCC as the mature evidence base while framing operative questions across hepatobiliary malignancies.

## 2 From anatomic to immuno-surgical resectability

We propose immuno-surgical resectability to describe a state in which surgery is technically feasible, liver reserve is adequate, immune-related toxicity is controlled, and the operative window matches tumor immune biology. It complements rather than replaces staging systems. Stable imaging after ICI therapy may conceal necrosis, fibrosis, and immune infiltrates, whereas impressive response can still harbor viable tumor. Surgical decisions should therefore integrate imaging, alpha-fetoprotein or CA19–9 kinetics, liver function, biliary infection, treatment toxicity, and, when available, pathological or circulating tumor DNA (ctDNA) indicators.

This concept differs by tumor type. In HCC, response must be judged against cirrhosis, portal hypertension, future liver remnant, and transplantation. In BTC, response must be translated into subtype-specific operability. Mass-forming ICC may become removable by hepatectomy and lymphadenectomy, whereas perihilar cholangiocarcinoma requires ductal-margin mapping, drainage of the future liver remnant, and feasible biliary or vascular reconstruction. Distal cholangiocarcinoma demands pancreaticoduodenectomy with negative radial and ductal margins, and gallbladder cancer requires exclusion of peritoneal or distant nodal disease before radical resection. Thus, in HCC surgery may consolidate immune control before liver reserve worsens; in BTC it must convert a likely R1/R2 situation into an anatomically realistic R0 operation (1, 2, 12–16).

Lymph nodes illustrate surgery as an immune intervention. Regional lymphadenectomy remains essential for BTC staging and removal of suspicious or anatomically indicated nodes. However, tumor-draining lymph nodes can reservoir stem-like tumor-reactive CD8-positive T cells that sustain PD-1/PD-L1 blockade (17). A recurrent BTC study linked excessive removal of non-metastatic tumor-draining nodes with impaired later immunotherapy efficacy (18). These findings support oncologically indicated lymphadenectomy, while cautioning against extended nodal clearance without evidence of benefit. Future BTC trials should report nodal stations, positive and negative nodes, nodal immune phenotype, postoperative infection, and subsequent immunotherapy response.

## 3 Surgical timing, washout, and transplantation

Surgical timing is now an immunological variable. Operating too early may forfeit systemic priming against micrometastatic disease; operating too late may allow resistant clones, vascular invasion, biliary sepsis, or hepatic decompensation. The safest window depends on tumor type, planned operation, drug class, liver reserve, and toxicity. ICIs require control of hepatitis, cholangitis-like injury, colitis, pneumonitis, endocrinopathy, myocarditis, and steroid dependence. Anti-VEGF therapy and TKIs add bleeding, thrombosis, hypertension, proteinuria, wound-healing, regeneration, and hepatic-injury concerns (19).

For BTC, the timing discussion must also account for biliary anatomy and infection control. Before conversion resection for ICC, the surgeon should confirm that response has made vascular inflow/outflow control and lymphadenectomy technically meaningful rather than merely smaller on imaging. In perihilar and distal cholangiocarcinoma, response should not obscure longitudinal ductal mapping, adequate drainage of the future liver remnant, nutritional optimization, and margin planning. In gallbladder cancer, apparent response should prompt renewed staging for peritoneal disease and distant nodal involvement before committing to radical resection. These considerations make hepatobiliary-wide decision making different from an HCC-only perioperative algorithm (16, 20, 21).

A practical timing discussion should answer five questions: Is the tumor still anatomically controllable, including vascular inflow/outflow and biliary drainage? Is liver reserve adequate for the planned hepatectomy? Have immune-related adverse events improved enough for major surgery and perioperative immunosuppression if needed? Is response deep enough to justify surgery now? Is the intent upfront cure, conversion, bridge to transplantation, or trial-based cytoreduction? This framing is more useful than one radiographic assessment (3–5, 19, 22).

Transplant candidates require particular caution. ICI-based downstaging or bridging can be considered when locoregional therapy is inadequate or unsafe, but should remain multidisciplinary. Individual patient-data analyses and multicenter cohorts show that longer ICI washout is associated with lower rejection risk; a washout approaching 50 days to 3 months is commonly favored when tumor control permits (10, 11, 23, 24). Pre-transplant ICI exposure therefore does not automatically preclude liver transplantation, but short washout should be treated as a surgical safety variable. The last ICI dose, anti-VEGF/TKI exposure, steroid history, donor timing, and rejection plan should be documented before listing.

**Figure 1:**
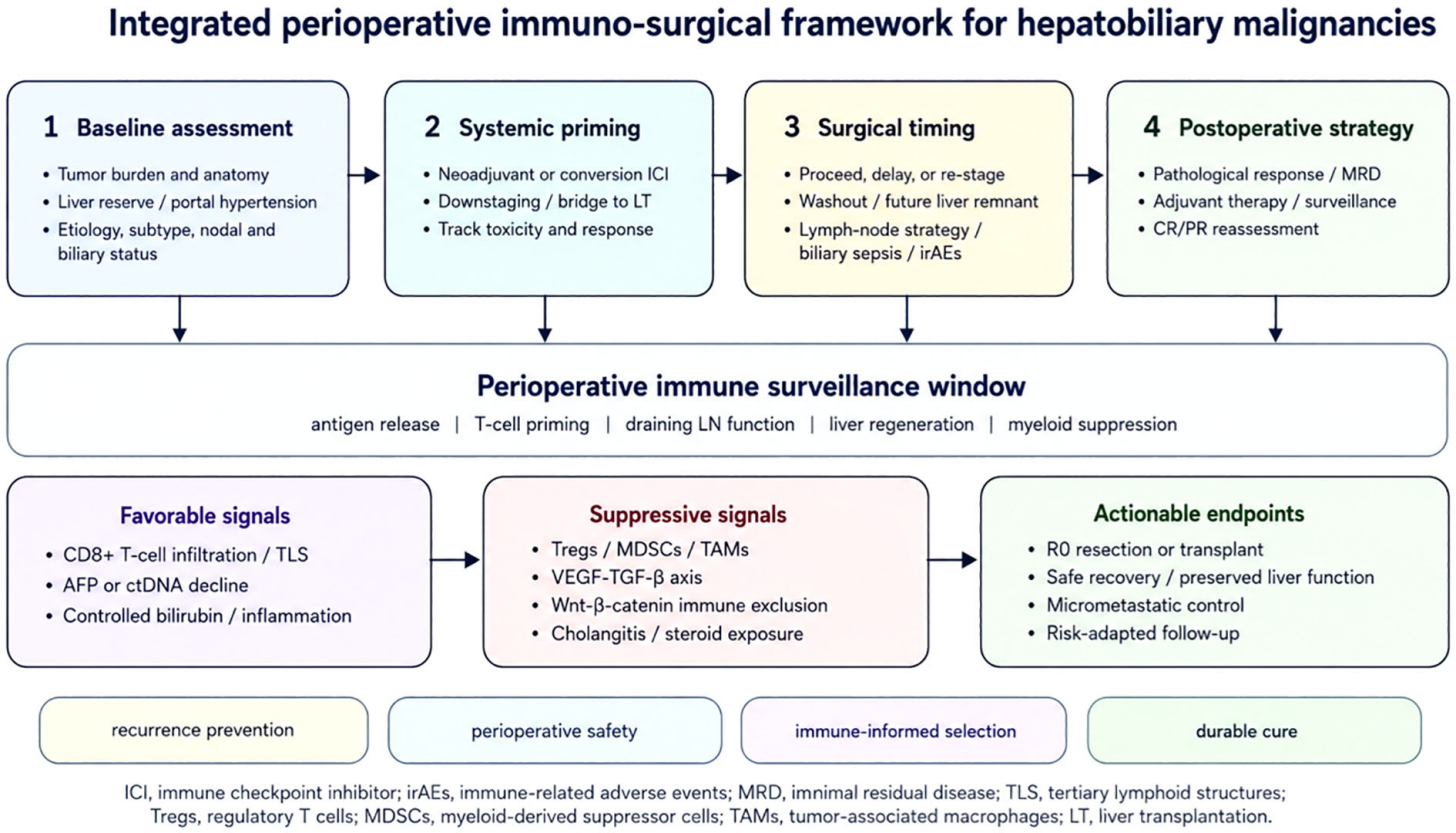
Integrated perioperative immuno-surgical framework for hepatobiliary malignancies.

Washout should not become a fixed calendar rule. Anti-VEGF agents may require longer intervals when wound healing, bleeding risk, or major vascular reconstruction are concerns. TKIs can often be stopped closer to surgery, but this depends on liver function, nutrition, toxicity, and operation magnitude. For transplantation, washout must be interpreted alongside donor availability and wait-list dropout. Final decisions should integrate pharmacology, toxicity recovery, disease tempo, and local logistics (10, 11, 23, 24). This pathway is summarized in **Figure 1**.

## 4 Treatment after CR/PR and recurrence prevention

Complete response (CR) or partial response (PR) after systemic therapy should trigger reassessment, not indefinite continuation. If R0 resection is feasible and liver reserve is adequate, surgery should be discussed before resistance, cholangitis, or decompensation develops. Radiographic CR is not pathological CR; residual masses after immunotherapy may contain viable tumor, and loss of enhancement does not guarantee eradication. For ICC or other BTC that becomes resectable, conversion surgery is rational only when R0 resection, adequate future liver remnant, and controlled biliary sepsis are achievable. For transplant candidates, response should prompt wait-list reassessment with explicit washout planning.

Patients with CR/PR can be grouped pragmatically. Operable patients who become resectable should undergo timely resection during the response window. Those still unresectable because of inadequate remnant liver, diffuse bilobar disease, or persistent vascular/biliary involvement should continue systemic therapy or trial-based locoregional combinations with repeat surgical review. Downstaged transplant candidates need reassessment of listing, timing, and rejection mitigation. Frail patients or those with borderline liver function may benefit more from non-operative disease control than from high-risk surgery driven by radiological enthusiasm alone (3–6, 10–16).

Postoperative treatment should be risk-adapted. In HCC, microvascular invasion, satellites, multifocality, poor differentiation, large size, narrow margins, and viable residual tumor indicate high risk. Atezolizumab plus bevacizumab after curative resection or ablation should be interpreted cautiously because updated IMbrave050 data did not confirm durable benefit (7, 8). Adjuvant sintilimab improved recurrence-free survival in a randomized phase II trial, but confirmatory phase III evidence is needed (9). In BTC, capecitabine remains the most established adjuvant option after resection, whereas ICI-chemotherapy combinations are standards for advanced disease and should be used perioperatively mainly in trials or selected conversion scenarios (12, 13, 20, 21, 25). Positive ctDNA, nodal disease, R1 margin, or viable residual tumor should prompt intensified surveillance, molecular profiling, and trial consideration rather than empiric escalation for all patients (22, 26–31).

ctDNA is particularly relevant because it offers a potential link between perioperative immune control and recurrence surveillance. It should be interpreted as a dynamic adjunct to imaging, pathology, tumor markers, and clinical status, not as a stand-alone indication for operation or systemic escalation. In HCC, postoperative ctDNA-based measures, including ctDNA-derived tumor mutational burden, have been associated with recurrence after resection, and a systematic review found ctDNA and circulating tumor cells to be promising MRD approaches while emphasizing assay heterogeneity and the lack of interventional validation (28, 29). In BTC, a multicenter stage I-III cohort showed ctDNA positivity during the MRD or surveillance window to be strongly associated with shorter relapse-free survival and earlier recurrence detection, and the STAMP extrahepatic cholangiocarcinoma biomarker cohort showed that longitudinal tumor-informed ctDNA predicted recurrence during adjuvant therapy and outperformed CA19–9 and CEA (30, 31). Reasonable research-oriented time points include baseline before systemic therapy, early post-resection after cell-free DNA noise settles, after adjuvant therapy, and surveillance. A positive result should prompt earlier hepatobiliary-protocol imaging, review of margins, nodal status and pathological response, molecular profiling, and trial discussion; a negative result may support surveillance or de-escalation only when integrated with low-risk pathology and preferably within prospective protocols.

Pathological response and MRD can further refine decisions. Major or complete pathological response after neoadjuvant therapy may support surveillance or de-escalated postoperative therapy when liver reserve is limited and ctDNA remains negative. Conversely, viable residual tumor, persistent or newly positive ctDNA, nodal metastasis, or narrow/R1 margins identify patients for whom adjuvant therapy or trial enrollment is more reasonable. In BTC, nodal involvement and margin status still dominate prognosis, but may increasingly be interpreted alongside immune remodeling and serial ctDNA dynamics (6–9, 25, 27–31). Key evidence-informed regimens, washout considerations, complications, and post-response decisions are summarized in **Table 1**.

**Table 1:** Evidence-informed perioperative regimens, washout considerations, complications, and post-response decisions.

Clinical setting	Regimen and dosage in key evidence	Evidence status	Washout/complications to manage	Decision after CR/PR or high-risk pathology
Resectable HCC; neoadjuvant or perioperative ICI	Nivolumab 240 mg IV every 2 weeks for 3 preoperative doses, then adjuvant nivolumab; or nivolumab plus ipilimumab in the phase II study (3)	Prospective randomized phase II; feasible in selected patients	No validated washout for hepatectomy; usually operate after planned preoperative cycle and recovery, commonly 2–4 weeks. Delay for grade >=2 hepatitis, pneumonitis, colitis, myocarditis, cholangitis-like injury, or steroid dependence (3, 26, 27)	If R0 resection is feasible after PR/SD, proceed during response window. Pathological CR/MPR supports surveillance or trial; viable high-risk tumor supports trial-oriented adjuvant strategy
Resectable HCC; neoadjuvant PD-1 blockade	Cemiplimab 350 mg IV every 3 weeks for 2 cycles before surgery (4)	Single-arm phase II; antitumor activity with acceptable perioperative safety	Assess liver tests and irAEs before major hepatectomy; do not equate radiographic CR with pathological CR	Proceed to resection when liver reserve and FLR are safe. Use pathology to guide surveillance intensity
Locally advanced HCC conversion	Cabozantinib 40 mg orally daily plus nivolumab 240 mg IV every 2 weeks (5)	Single-arm phase Ib conversion strategy	Hold TKI before major surgery, typically 1–2 weeks depending on toxicity; manage hypertension, proteinuria, bleeding risk, wound healing, diarrhea, and hepatic dysfunction	Convert to resection only if R0 margins and adequate FLR are achievable; persistent progression suggests nonoperative systemic strategy
HCC after curative resection or ablation; high recurrence risk	Atezolizumab 1200 mg IV plus bevacizumab 15 mg/kg IV every 3 weeks for up to 12 months in IMbrave050 (7, 8)	Phase III initially positive for RFS; updated analysis weakened support for routine use	Postoperative start only after wound healing and recovery. Monitor bleeding, hypertension, proteinuria, thrombosis, hepatic dysfunction, and irAEs	Not a universal standard. Consider only in high-risk patients after MDT discussion or trial; observation reasonable after low-risk pathology or pCR/MRD negativity
Resected HCC with microvascular invasion; adjuvant PD-1	Sintilimab 200 mg IV every 3 weeks for 8 cycles (9)	Randomized phase II; confirmatory phase III needed	Begin after adequate surgical recovery; monitor immune hepatitis in cirrhosis and HBV reactivation risk with antiviral control	High-risk pathology may justify trial or region-specific discussion; avoid routine escalation for all resected patients
HCC transplant candidates; downstaging/bridge	ICI-based therapy alone or with anti-VEGF/TKI/liver-directed therapy; regimens vary (10, 11)	Retrospective cohorts and individual-patient-data meta-analysis; no definitive RCT	Rejection risk rises with short washout. Prefer approximately 3 months when feasible; document last ICI dose, anti-VEGF/TKI exposure, steroid use, donor timing, and rejection plan	After CR/PR, reassess transplant criteria rather than continuing ICI indefinitely. Proceed only after informed washout and center-specific risk review
Advanced BTC first line; potential preoperative reassessment if exceptional response	Durvalumab 1500 mg IV day 1 every 3 weeks plus gemcitabine 1000 mg/m2 and cisplatin 25 mg/m2 days 1 and 8 for up to 8 cycles, then durvalumab 1500 mg every 4 weeks (12)	Phase III standard for advanced BTC	Before surgery allow marrow, renal, hepatic, and biliary recovery; control cholangitis and stent dysfunction; assess immune cholangitis vs obstruction (12, 26, 27)	CR/PR may justify conversion surgery if R0 resection and safe FLR are possible; otherwise continue systemic therapy or maintenance
Advanced BTC first line; alternative ICI-chemotherapy	Pembrolizumab 200 mg IV every 3 weeks plus gemcitabine 1000 mg/m2 and cisplatin 25 mg/m2 days 1 and 8 (13)	Phase III standard for advanced BTC	Same cytotoxic and ICI precautions; avoid surgery during active infection, biliary obstruction, or unresolved irAE	Conversion surgery requires MDT review; radiographic response alone is insufficient
Borderline or initially unresectable perihilar/distal cholangiocarcinoma or gallbladder cancer after major systemic response	No established neoadjuvant ICI standard. If durvalumab or pembrolizumab plus gemcitabine-cisplatin produces exceptional response in advanced BTC, repeat subtype-specific surgical assessment (12, 13, 16, 20, 21)	Advanced-disease phase III regimens support systemic control; conversion surgery evidence remains limited and should be MDT- and trial-oriented	Control cholangitis, stent dysfunction, bilirubin, nutrition, renal function, cytopenia, neuropathy, and irAEs. Plan ductal, vascular, pancreaticoduodenectomy, or gallbladder-specific margins before stopping therapy	Proceed only if R0 resection, safe FLR or pancreatic remnant, and anatomically justified lymphadenectomy are feasible. Occult peritoneal, distant nodal, or persistent vascularly unreconstructable disease favors systemic therapy or trial
Advanced or unresectable ICC; TKI + GEMOX + anti-PD-1	Toripalimab 240 mg IV every 3 weeks plus lenvatinib 8 mg orally daily and GEMOX: gemcitabine 1 g/m2 days 1 and 8 plus oxaliplatin 85 mg/m2 every 3 weeks (14)	Single-center, single-arm phase II (14); high response but not yet standard perioperative care	Hold TKI before surgery; allow cytopenia, neuropathy, bilirubin, cholangitis, and nutrition to recover. Consider 3–4 weeks after last chemotherapy if clinically stable	For major PR in ICC, consider conversion resection only if R0 is realistic, FLR is adequate, and nodal/distant disease is controlled. Persistent nodal or distant disease favors systemic therapy or trial
Resected BTC; adjuvant standard	Capecitabine 1250 mg/m2 orally twice daily on days 1–14 every 21 days for 8 cycles (25)	Randomized phase III evidence; still the reference adjuvant option	Start after postoperative recovery, typically within 16 weeks in BILCAP. Manage hand-foot syndrome, diarrhea, mucositis, cytopenias, renal function, and bilirubin	Use after resected cholangiocarcinoma or gallbladder cancer when tolerated; ICI-based adjuvant therapy should preferably be trial-based

BTC, biliary tract cancer; CR, complete response; ctDNA, circulating tumor DNA; dCCA, distal cholangiocarcinoma; eCCA, extrahepatic cholangiocarcinoma; FLR, future liver remnant; GEMOX, gemcitabine-oxaliplatin; HCC, hepatocellular carcinoma; ICC, intrahepatic cholangiocarcinoma; ICI, immune checkpoint inhibitor; irAE, immune-related adverse event; MDT, multidisciplinary team; MPR, major pathological response; MRD, minimal residual disease; pCCA, perihilar cholangiocarcinoma; PR, partial response; RFS, recurrence-free survival; TKI, tyrosine kinase inhibitor. Suggested washout intervals are pragmatic decision points and must be individualized according to drug half-life, operation type, liver function, biliary sepsis, toxicity, and transplant urgency.

## 5 Evidence base and literature-search approach

To support this Opinion, PubMed and ClinicalTrials.gov were searched to 12 June 2026 using combinations of hepatocellular carcinoma, cholangiocarcinoma, biliary tract cancer, gallbladder cancer, immunotherapy, immune checkpoint inhibitor, neoadjuvant, adjuvant, conversion therapy, liver transplantation, washout, lymph-node dissection, ctDNA, and recurrence. Priority was given to randomized trials, prospective phase I/II studies, multicenter cohorts, guidelines, and translational studies published from 2021 onward. Older landmark studies were retained only when they remain the standard source for current adjuvant or surgical practice.

## 6 Discussion

The practical implication is that hepatobiliary surgeons should be involved before systemic therapy begins in potentially operable patients. Delayed referral can miss the conversion window, compromise future liver-remnant planning, or expose transplant candidates to avoidable rejection risk. Operability should not be decided by RECIST response alone; it should combine response depth, liver reserve, biliary status, immune toxicity, nodal strategy, MRD information when available, and the intended surgical goal. Biology also matters: NASH-related HCC and immune-evasive microenvironments may blunt ICI benefit, while ICC, perihilar cholangiocarcinoma, distal cholangiocarcinoma, and gallbladder cancer differ in stromal architecture, biliary contamination, mutational drivers, and patterns of lymphatic spread. These differences complicate direct extrapolation from HCC to all hepatobiliary malignancies (32–34).

Uncertainties should temper enthusiasm. Most perioperative HCC data come from small phase I/II studies, and BTC conversion evidence is still dominated by single-center cohorts rather than randomized surgery-specific trials. Pathological response definitions are not standardized across HCC and BTC. Biomarkers such as ctDNA, tertiary lymphoid structures, and spatial immune-cell architecture are promising, but should not be used as isolated decision tools outside validated protocols. Even so, immunotherapy makes immune biology, toxicity recovery, subtype-specific anatomy, and recurrence risk as central to operative planning as anatomy itself (6, 14, 15, 27–31).

Future perioperative trials should report surgical endpoints with the same rigor as oncological endpoints: R0 rate, major hepatectomy rate, lymph-node yield and stations, bile leak, post-hepatectomy liver failure, infection, transfusion, readmission, steroid exposure, interval from last systemic dose to surgery, pathological response, ctDNA assay type and sampling time points, ctDNA kinetics, and recurrence pattern. BTC studies should stratify by anatomical subtype, nodal burden, drainage, infection, reconstruction, and conversion intent, because immunochemotherapy has different operative implications for mass-forming ICC, perihilar or distal cholangiocarcinoma, and gallbladder cancer (14–16, 20, 21, 30, 31). The key task is to identify patients in whom surgery consolidates immune control, and to avoid operations that may interrupt that balance. Hepatobiliary surgery should therefore move from anatomic resectability alone toward immuno-surgical resectability, preserving technical curability and durable antitumor immune surveillance.

## References

[1] Singal AG Llovet JM Yarchoan M Mehta N Heimbach JK Dawson LA . AASLD practice guidance on prevention, diagnosis, and treatment of hepatocellular carcinoma. Hepatology. (2023) 78:1922–65. doi: 10.1097/HEP.0000000000000466 37199193 PMC10663390

[2] Vogel A Chan SL Dawson LA Kelley RK Llovet JM Meyer T . Hepatocellular carcinoma: ESMO clinical practice guideline for diagnosis, treatment and follow-up. Ann Oncol. (2025) 36:491–506. doi: 10.1016/j.annonc.2025.02.006 39986353

[3] Kaseb AO Hasanov E Cao HST Xiao L Vauthey JN Lee SS . Perioperative nivolumab monotherapy versus nivolumab plus ipilimumab in resectable hepatocellular carcinoma: a randomised, open-label, phase 2 trial. Lancet Gastroenterol Hepatol. (2022) 7:208–18. doi: 10.1016/S2468-1253(21)00427-1 35065057 PMC8840977

[4] Marron TU Fiel MI Hamon P Fiaschi N Kim E Ward SC . Neoadjuvant cemiplimab for resectable hepatocellular carcinoma: a single-arm, open-label, phase 2 trial. Lancet Gastroenterol Hepatol. (2022) 7:219–29. doi: 10.1016/S2468-1253(21)00385-X 35065058 PMC9901534

[5] Ho WJ Zhu Q Durham J Popovic A Xavier S Leatherman J . Neoadjuvant cabozantinib and nivolumab converts locally advanced HCC into resectable disease with enhanced antitumor immunity. Nat Cancer. (2021) 2:891–903. doi: 10.1038/s43018-021-00234-4 34796337 PMC8594857

[6] D'Alessio A Stefanini B Blanter J Adegbite B Crowley F Yip V . Pathological response following neoadjuvant immune checkpoint inhibitors in patients with hepatocellular carcinoma: a cross-trial, patient-level analysis. Lancet Oncol. (2024) 25:1465–75. doi: 10.1016/S1470-2045(24)00457-1 39437804 PMC12040480

[7] Qin S Chen M Cheng AL Kaseb AO Kudo M Lee HC . Atezolizumab plus bevacizumab versus active surveillance in patients with resected or ablated high-risk hepatocellular carcinoma (IMbrave050): a randomised, open-label, multicentre, phase 3 trial. Lancet. (2023) 402:1835–47. doi: 10.1016/S0140-6736(23)01796-8 37871608

[8] Yopp A Chen M Cheng AL Kaseb A Kudo M Lee HC . Updated data from IMbrave050: Adjuvant atezolizumab plus bevacizumab for high-risk hepatocellular carcinoma. J Hepatol. (2026) 84:1102–11. doi: 10.1016/j.jhep.2026.01.006 41580093

[9] Wang K Xiang YJ Yu HM Cheng YQ Liu ZH Qin YY . Adjuvant sintilimab in resected high-risk hepatocellular carcinoma: a randomized, controlled, phase 2 trial. Nat Med. (2024) 30:708–15. doi: 10.1038/s41591-023-02786-7 38242982

[10] Rezaee-Zavareh MS Yeo YH Wang T Guo Z Tabrizian P Ward SC . Impact of pre-transplant immune checkpoint inhibitor use on post-transplant outcomes in HCC: a systematic review and individual patient data meta-analysis. J Hepatol. (2025) 82:107–19. doi: 10.1016/j.jhep.2024.06.042 38996924 PMC11655254

[11] Li M Bhoori S Mehta N Mazzaferro V . Immunotherapy for hepatocellular carcinoma: The next evolution in expanding access to liver transplantation. J Hepatol. (2024) 81:743–55. doi: 10.1016/j.jhep.2024.05.037 38848767

[12] Oh DY He AR Bouattour M Okusaka T Qin S Chen LT . Durvalumab or placebo plus gemcitabine and cisplatin in participants with advanced biliary tract cancer (TOPAZ-1): updated overall survival from a randomised phase 3 study. Lancet Gastroenterol Hepatol. (2024) 9:694–704. doi: 10.1016/S2468-1253(24)00095-5 38823398

[13] Kelley RK Ueno M Yoo C Finn RS Furuse J Ren Z . Pembrolizumab in combination with gemcitabine and cisplatin compared with gemcitabine and cisplatin alone for patients with advanced biliary tract cancer (KEYNOTE-966): a randomised, double-blind, placebo-controlled, phase 3 trial. Lancet. (2023) 401:1853–65. doi: 10.1016/S0140-6736(23)00727-4 37075781

[14] Shi GM Huang XY Wu D Sun HC Liang F Ji Y . Toripalimab combined with lenvatinib and GEMOX is a promising regimen as first-line treatment for advanced intrahepatic cholangiocarcinoma: a single-center, single-arm, phase 2 study. Signal Transduct Target Ther. (2023) 8:106. doi: 10.1038/s41392-023-01317-7 36928584 PMC10020443

[15] Zhu C Xue J Wang Y Wang S Zhang N Wang Y . Efficacy and safety of lenvatinib combined with PD-1/PD-L1 inhibitors plus Gemox chemotherapy in advanced biliary tract cancer. Front Immunol. (2023) 14:1109292. doi: 10.3389/fimmu.2023.1109292 36742297 PMC9889821

[16] Vogel A Ducreux M ESMO Guidelines Committee . ESMO clinical practice guideline interim update on the management of biliary tract cancer. ESMO Open. (2025) 10:104003. doi: 10.1016/j.esmoop.2024.104003 39864891 PMC11846563

[17] Huang Q Wu X Wang Z Chen X Wang L Lu Y . The primordial differentiation of tumor-specific memory CD8+ T cells as bona fide responders to PD-1/PD-L1 blockade in draining lymph nodes. Cell. (2022) 185:4049–4066.e25. doi: 10.1016/j.cell.2022.09.020 36208623

[18] Long Y An B Li Q Geng Y Zhou Y Geng Z . Excessive dissection of non-metastatic tumor-draining lymph nodes impairs immunotherapy efficacy in recurrent biliary tract cancer. Clin Cancer Res. (2025) 32(12):2467–78. doi: 10.1158/1078-0432.CCR-25-3296 41460246

[19] Bezu L Akçal Öksüz D Bell M Buggy D Diaz-Cambronero O Enlund M . Perioperative immunosuppressive factors during cancer surgery: an updated review. Cancers (Basel). (2024) 16:2304. doi: 10.3390/cancers16132304 39001366 PMC11240822

[20] Vogel A Bridgewater J Edeline J Kelley RK Klümpen HJ Malka D . Biliary tract cancer: ESMO clinical practice guideline for diagnosis, treatment and follow-up. Ann Oncol. (2023) 34:127–40. doi: 10.1016/j.annonc.2022.10.506 36372281

[21] Casak SJ Kumar V Song C Yuan M Amatya AK Cheng J . FDA approval summary: Durvalumab and pembrolizumab, immune checkpoint inhibitors for the treatment of biliary tract cancer. Clin Cancer Res. (2024) 30:3371–7. doi: 10.1158/1078-0432.CCR-24-0517 38856639 PMC11326973

[22] Schneider BJ Naidoo J Santomasso BD Lacchetti C Adkins S Anadkat M . Management of immune-related adverse events in patients treated with immune checkpoint inhibitor therapy: ASCO guideline update. J Clin Oncol. (2021) 39:4073–126. doi: 10.1200/JCO.21.01440 34724392

[23] Guo Z Liu Y Ling Q Xu L Wang T Zhu J . Pretransplant use of immune checkpoint inhibitors for hepatocellular carcinoma: a multicenter, retrospective cohort study. Am J Transplant. (2024) 24:1837–56. doi: 10.1016/j.ajt.2024.04.007 38642712

[24] Moeckli B Wassmer CH El Hajji S Kumar R Rodrigues Ribeiro J Tabrizian P . Determining safe washout period for immune checkpoint inhibitors prior to liver transplantation: an international retrospective cohort study. Hepatology. (2025) 82:1122–37. doi: 10.1097/HEP.0000000000001289 40042053

[25] Primrose JN Fox RP Palmer DH Malik HZ Prasad R Mirza D . Capecitabine compared with observation in resected biliary tract cancer (BILCAP): a randomised, controlled, multicentre, phase 3 study. Lancet Oncol. (2019) 20:663–73. doi: 10.1016/S1470-2045(18)30915-X 30922733

[26] Pi B Wang J Tong Y Yang Q Lv F Yu Y . Immune-related cholangitis induced by immune checkpoint inhibitors: a systematic review of clinical features and management. Eur J Gastroenterol Hepatol. (2021) 33:e858–67. doi: 10.1097/MEG.0000000000002280 34482313 PMC8734631

[27] Xu Y Cai J Zhong K Wen Y Cai L He G . Plasma-only circulating tumor DNA analysis detects minimal residual disease and predicts early relapse in hepatocellular carcinoma patients undergoing curative resection. Front Oncol. (2023) 13:1119744. doi: 10.3389/fonc.2023.1119744 36959801 PMC10028131

[28] Wehrle CJ Hong H Kamath S Schlegel A Fujiki M Hashimoto K . Tumor mutational burden from circulating tumor DNA predicts recurrence of hepatocellular carcinoma after resection: an emerging biomarker for surveillance. Ann Surg. (2024) 280:504–13. doi: 10.1097/SLA.0000000000006386 38860385

[29] Galli E Patelli G Villa F Gri N Mazzarelli C Mangoni I . Circulating blood biomarkers for minimal residual disease in hepatocellular carcinoma: a systematic review. Cancer Treat Rev. (2025) 135:102908. doi: 10.1016/j.ctrv.2025.102908 40058162

[30] Yu J He AR Ouf M Mehta R Anaya DA Denbo J . Detecting early recurrence with circulating tumor DNA in stage I-III biliary tract cancer after curative resection. JCO Precis Oncol. (2025) 9:e2400443. doi: 10.1200/PO-24-00443 39772829 PMC11723488

[31] Yoo C Jeong H Jeong JH Kim KP Lee S Ryoo BY . Circulating tumor DNA status and dynamics predict recurrence in patients with resected extrahepatic cholangiocarcinoma. J Hepatol. (2025) 82:861–70. doi: 10.1016/j.jhep.2024.10.043 39532185

[32] Pfister D Núñez NG Pinyol R Govaere O Pinter M Szydlowska M . NASH limits anti-tumour surveillance in immunotherapy-treated HCC. Nature. (2021) 592:450–6. doi: 10.1038/s41586-021-03362-0 33762733 PMC8046670

[33] Chen C Wang Z Ding Y Qin Y . Tumor microenvironment-mediated immune evasion in hepatocellular carcinoma. Front Immunol. (2023) 14:1133308. doi: 10.3389/fimmu.2023.1133308 36845131 PMC9950271

[34] Giraud J Chalopin D Blanc JF Saleh M . Hepatocellular carcinoma immune landscape and the potential of immunotherapies. Front Immunol. (2021) 12:655697. doi: 10.3389/fimmu.2021.655697 33815418 PMC8012774

